# Mechanical, sensory, and consumer evaluation of ketogenic, gluten‐free breads

**DOI:** 10.1002/fsn3.2308

**Published:** 2021-05-04

**Authors:** Rachel Gillespie, Gene J. Ahlborn

**Affiliations:** ^1^ Brigham Young University Provo UT USA

**Keywords:** almond flour, bread, gluten‐free, ketogenic, oat bran fiber

## Abstract

Ketogenic, gluten‐free breads comprised of almond flour, oat bran fiber, or combinations of both were compared. The textural properties, sensory attributes, and consumer acceptance were analyzed on each bread containing 100% almond flour (AF), 66.7% almond flour with 33.3% oat bran fiber (AOB), 66.7% oat bran fiber with 33.3% almond flour (OBA), and 100% oat bran fiber (OB). AF and AOB breads had a more open crumb structure composed of cells between 1–4 mm^2^. OBA and OB had a significantly dense crumb pattern made up of more cells less than one millimeter squared. Quantitative–descriptive analysis (QDA) and consumer acceptance testing was conducted 24 hr after baking and mechanical endpoints were evaluated 24, 72, and 120 hr after baking. AF and AOB breads were preferred over OBA and OB breads in QDA evaluation and consumer acceptance scores. Greater percentages of oat bran fiber resulted in a bread that was less moist, firmer in texture, and chewier with trained panelists. In both sensory evaluations, higher amounts of almond flour resulted in higher values in eggy flavor while increased amounts of oat bran fiber correlated with higher values in earthy flavor. Mechanical testing identified higher percentages of almond flour resulted in bread that was less firm and less chewy. Over time, all variations with almond flour became softer and less chewy, while the OB bread increased in firmness. Sensory cohesiveness did not correlate with the mechanical equivalent, identifying a need to re‐evaluate the parameters used to calculate this objective endpoint.

## Graphical abstract

Textural properties, sensory attributes, and consumer acceptance were analyzed for ketogenic, gluten‐free breads comprised of almond flour, oat bran fiber, or combinations of both. Higher ratios of almond flour to oat bran fiber resulted in a bread with better structure, flavor and texture which was preferred by trained panelist and consumers.

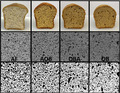

## 1 INTRODUCTION

Bread has been a staple for thousands of years and is consumed daily in many countries. However, traditional breads are not acceptable to consume for individuals on a low carbohydrate diet as they typically average 15–20 g of carbohydrates per serving. Low carbohydrate options are becoming more popular, particularly for those following a ketogenic diet. Ketogenic diets are a growing trend for both weight loss and as a means of treating diabetes (Bolla et al., 2019; Churuangsuk et al., 2018; Merrill et al., 2020; Westman et al., 2018; Yancy et al., 2019). Studies also indicate a ketogenic diet could be beneficial in treating and reducing the effect of neurological disorders, including Autism, Alzheimer's, Parkinson's disease, and as an alternative for treating/reducing seizures (Castro et al., 2015; Davis et al., 2020; Li et al., 2020).

Ketogenic diets involve eating a carbohydrate‐restricted diet, requiring the body to burn fat as a primary energy source. Ketogenic diets are higher in fat and protein, with the protein source typically being derived from animal sources. The reduction in carbohydrates puts the body into the metabolic state of ketosis, resulting in lower blood sugar and decreased insulin requirement. There are multiple health benefits, as well as substantial health concerns, associated with inducing a state of ketosis, especially as an extended lifestyle option. This paper will not address all the specifics around benefits and concerns with ketogenic diets as its focus is to address properties of ketogenic bread and will, however, include a sample of additional papers which are available in the literature for further review (Davis et al., 2020; Johnston et al., 2006; Kossoff & McGrogan, 2005; Kraft & Westman, 2009; Li & Heber, 2020; O'Neill & Raggi, 2020; Ruiz Herrero et al., 2020).

It is relevant to briefly note associations between neurological disorders in both ketogenic and gluten‐free diets. It was postulated that Celiac disease may be correlated to an increase in psychiatric illness, including neurological issues such as peripheral neuropathy (Cooke & Smith, 1966), myopathy (Binder et al., 1967), cerebellar ataxia (Hermaszewski et al., 1991), cerebral atrophy, and dementia (Collin et al., 1991), an increase in epilepsy (Bouquet, 1992) and other diseases. Researchers have previously noted an association between schizophrenia and celiac disease, an immune‐mediated enteropathy that is triggered by the ingestion of gluten‐containing grains (Kalaydjian et al., 2006). A sampling of papers on gluten‐related conditions and diseases are available for review (Aziz et al., 2016; Aziz & Sanders, 2012; Lerner et al., 2019; Mansueto et al., 2014; Rotondi Aufiero et al., 2018; Singh et al., 2018; Tanveer & Ahmed, 2019; Włodarek, 2019).

Individuals following a gluten‐free diet avoid the consumption of wheat and grains that contain gluten. Gluten is a crucial part of the development of bread because it contributes to the structure. Significant research has been conducted using non‐gluten‐containing flours and starches including rice, tapioca, potato, corn, and oat among others, to create a suitable gluten bread substitute. However, as these flours and starches contain a high amount of carbohydrates, they are not viable options for use in a ketogenic bread product. With the gluten‐free market being projected to reach over $42 million dollars by 2027, and with the additional demand for ketogenic products, there are increasing demands for bread options that are both ketogenic and gluten‐free.

Suitable flours for a ketogenic, gluten‐free product, should have less than five percent available carbohydrates. Due to the higher fat and protein contents, and limited or non‐digestible carbohydrates (fiber), flours made from nuts, seeds, soy, and some legumes are ideally suited to satisfy consumer needs. However, not all of these flours are acceptable. For example, cashew flour contains excessive carbohydrates to meet ketogenic requirements. Additionally, the flavors in coconut, chickpea, flaxseed, and soy flours were identified as overwhelmingly strong during early exploration. In investigational studies, other potential flours such as flax and sesame meal produced inferior crumb structures (unpublished data). Almond flour was identified as the most suitable candidate for these breads due to its nutritional and sensory attributes. Oat bran fiber was also identified as a strong candidate and showed more promise as a primary ingredient for its ability to provide an improved structure while adhering to the low net carbohydrate requirements. Psyllium husk also met the gluten‐free and ketogenic criteria, providing unique characteristics to early prototypes. However, as a primary ingredient psyllium husk alone resulted in denser, heavy bread, and functioned better as an adjuvant for almond flour and oat bran fiber (data are not shown).

As such, the purpose of this study was to evaluate ketogenic, gluten‐free breads formulated with varying levels of almond flour and oat bran fiber through sensory and mechanical means.

## 2 MATERIALS AND METHODS

### 2.1 Bread making and materials

Breads tested were developed in the Brigham Young University Bread and Dairy Laboratory following extensive optimization of multiple formulas and preliminary sensory preference evaluations. Formulations for tested breads are given in Table 1. Specified ratios of almond flour (Costco Wholesale) comprised of 75% fat, 13.3% protein, 7% dietary fiber and <5% simple carbohydrate, and 100% oat bran fiber (NuNaturals Inc.) were combined with 8 g baking powder (Clabber Girl Corp), 1 g salt (NaCl), 0.5 g nutritional yeast (Bragg Live Food Products) 15 g whole psyllium husk (Now Foods), and set aside to be later combined with wet ingredients. Two eggs at room temperature were beaten with the whisk attachment in an electric mixer (Model KSM180 QHSD; KitchenAid). After which, 25 g of unsalted butter was melted, added to 50 g of softened cream cheese and mixed for four minutes until homogeneous. Half of the dry mixture was added to the wet mixture and beat for one minute until fully blended, after which the remaining dry ingredients were fully incorporated. Fifty ml boiling water and 7.2 g of apple cider vinegar (Bragg Live Food Products) were combined and added to the dough mixture. The bowl was covered and let stand for 15 min to allow all ingredients to properly hydrate. Dough (290 g) was placed into pup loaf pans (14.6 cm × 7.62 cm) lined with parchment paper and baked for 50 min at 176°C in a rotary oven (Model 12/24‐SS; National Manufacturing). After 1 hr of cooling, bread was sliced with the Omcan 44,247 Commercial Bread Slicer (Model SB‐CN‐0013) and placed in plastic bags for subsequent testing.

**TABLE 1:** Ketogenic, gluten‐free bread formulations and nutritional data

Ingredient	100% Almond Flour	66.7% Almond Flour/ 33.3% Oat Fiber	66.7% Oat Fiber/33.3% Almond Flour	100% Oat Fiber
	(AF)	(AOB)	(OBA)	(OB)
Oat bran fiber	0 g	16.6 g	33.3 g	50 g
Almond flour	50 g	33.3 g	16.6 g	0 g
Eggs	100 g s	100 g	100 g s	100 g
Butter	25 g	25 g	25 g	25 g
Cream cheese	50 g	50 g	50 g	50 g
Psyllium husk	15 g	15 g	15 g	15 g
Backing powder	8 g	8 g	8 g	8 g
Boiling water	50 g	50 g	50 g	50 g
Vinegar	7.2 g	7.2 g	7.2 g	7.2 g
Salt	1 g	1 g	1 g	1 g
Nutritional yeast	0.5 g	0.5 g	0.5 g	0.5 g
Nutritional data	Approximate percentages ±0.5%			
Fat %	80.5%	80.7%	81.0%	80.5%
Protein %	12.7%	12.5%	12.0%	11.5%
Net carbs %	6.7%	6.3%	6.5%	6.8%

### 2.2 Loaf volume and moisture

Loaf volume was conducted by the Rapeseed Displacement method 10–05.01 and moisture content was conducted by the two‐stage bread moisture method 44–15.02 as described by the American Association of Cereal Chemistry (AACC, 2000).

### 2.3 Crumb quality

Crumb quality was evaluated through digital image analysis (DIA) using ImageJ (National Institute of Health). Samples were cut as described above. Images were scanned using a flatbed scanner (Laser Jet Pro MFP; Hewlett Packard), analyzed according to the procedure described by Rosales‐Juarez et al. (2008), and photographed with an iPhone 11 (Apple Inc.) with a dual‐lens 12 MP camera (26 mm f/1.8; 13 mm f/2.4). Photos were converted to 8‐bit images, measurement scales calibrated, and cells measured manually and through Threshold calculations as described (SMSTechEdu, 2017). Four slices from different loaves were analyzed for cell size and frequency per centimeter squared.

### 2.4 Mechanical testing

Bread crumb firmness, cohesion, adhesion, springiness, and chewiness were measured on a TA‐XT2 texture analyzer (Texture Technologies Corp.) according to AACC method 74–09 modified with a 10 N load cell and a probe speed of 1.7 mm/sec, set 10 mm from the bread surface using a with a TA‐4 acrylic cylinder (25‐mm diameter, 35‐mm tall) probe. Applications software (Texture Exponent 32, V6.1.13.0, Stable Micro Systems Ltd.) and system macros were applied without modification. Texture measurement (12 values) was performed using three center samples from four loaves per treatment. Two bread slices were combined to make a 25‐mm thick sample for compression to 60% deformation. After initial deformation, samples rested for 5 s, followed by a second compression cycle. Firmness was a measurement of the maximum force of the first compression. Cohesiveness was the positive area of work of the second compression divided by that of the first compression. Springiness was expressed as the ratio of the distance of the detected height during the second compression and the original compression distance. Adhesiveness was measured as the negative area of compression one. Chewiness is expressed as firmness multiplied by cohesiveness and springiness. Samples were tested 24, 72, and 120 hr after baking.

### 2.5 Sensory evaluation through quantitative descriptive analysis

Twelve trained panelists, all regular consumers of bread, met weekly for a twelve‐week period to establish descriptor attributes and terminology around bread attributes. Reference standards and performance evaluations were conducted as described by Ahlborn and colleagues (Ahlborn et al., 2005). Attributes for analysis were developed and refined through consensus and ballot methods, facilitated through group discussion. Final attributes selected for evaluation at 24 hr post‐baking were: moistness, adhesion, cohesion, yeast flavor, buttery flavor, earthy flavor, eggy flavor, and chewiness. Texture attributes followed the definitions established by Carson and colleagues (Carson et al., 2000).

### 2.6 Sensory evaluation through consumer acceptance

Consumer preference/acceptance of breads was conducted at Brigham Young University Sensory Laboratory. Recruited participants (104 people, 48% female and 52% male) were divided into two groups, with one being informed they were evaluating a ketogenic, gluten‐free bread. The second group was just informed they would be evaluating bread. Following their evaluation, participants of the second group were informed the breads were ketogenic and gluten‐free and asked to re‐evaluate their impression of breads. Every panelist received samples by means of a pass‐through compartment following a monadic sequential order. Sample were served on separate foam plates with distilled water and crackers for pallet cleansing between samples. Attributes selected for evaluation were: appearance, aroma, flavor, texture, aftertaste, moistness, chewiness, color, yeasty flavor, earthy flavor, and eggy flavor. Questions were delivered on a computer screen using Compusense Cloud® (Compusense, Inc.) software instructing panelists to evaluate samples one at a time. Sensory evaluation was approved through the Brigham Young University Institutional Review Board and panelists provided informed consent prior to participation.

### 2.7 Statistical analysis

Data were analyzed using JMP® Pro 15.0 (SAS, Institute Inc.). Treatment effects were compared through Least Square Means with Tukey‐Kramer grouping to differentiate treatment effects and significant differences determined at α ≤ .05. Multiple linear regression was conducted to determine significant of the interaction between variables and Pearson's correlation coefficients were applied to determine if positive or negative correlations existed between the different terms analyzed. Consumer acceptance data were analyzed using analysis of variance with Tukey's HSD, except for ranking data, which were analyzed using Friedman Analysis of Ranking. Significance was set at a *p*‐value < .05.

## 3 RESULTS AND DISCUSSION

### 3.1 Loaf volume and crumb quality

**FIGURE 1:**
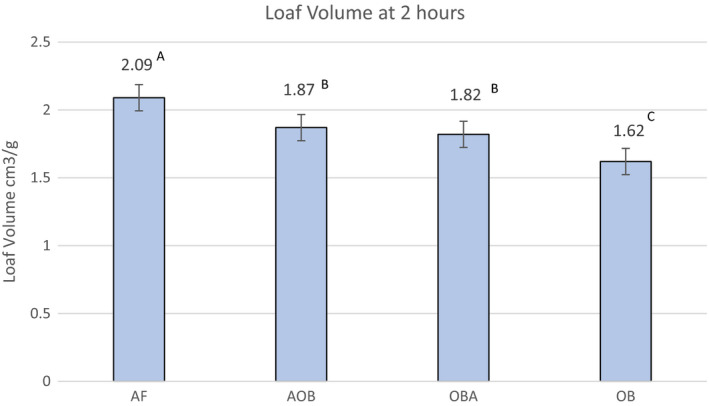
Loaf volumes of ketogenic, gluten‐free breads 2 hr after baking. Values with common letters are not significantly different (*p* < .05)

Loaf volume was greatest in AF (Figure 1) and no difference was observed between volumes of AOB and OBA, with OB having the lowest volume in cubic centimeter/gram. Visual inspection also identified AF as having the most volume. In the absence of gluten, egg albumin properties contribute greatly to forming the cell structure and supporting gas retention (Ziobro et al., 2016).

**FIGURE 2:**
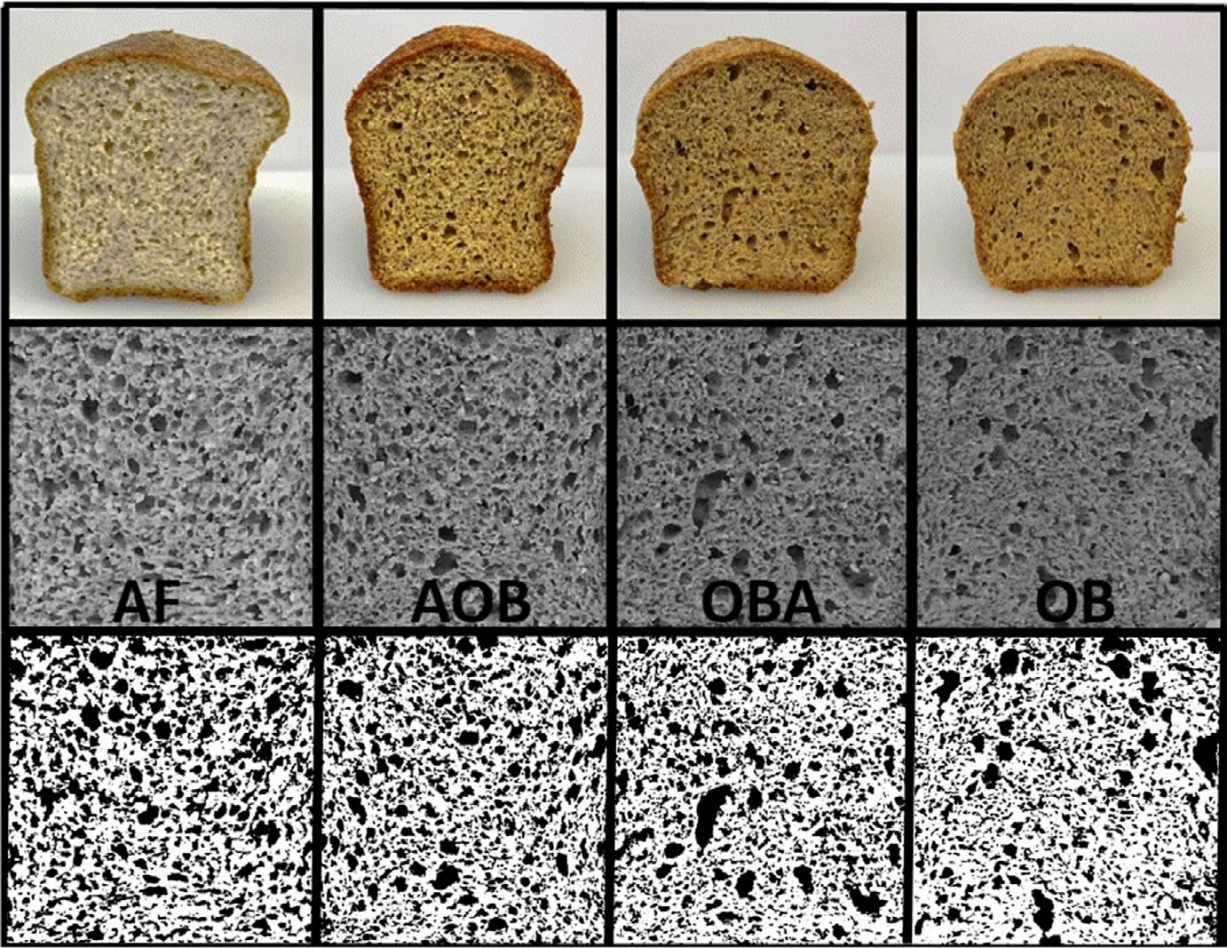
Ketogenic, gluten‐free bread photos from iPhone (above) and 8‐bit modified images for ImageJ crumb analysis (below)

Scanned and photographed images were analyzed with all methods described. Photographs (Figure 2) were of better quality and resolution than scanned images (data not shown). Likewise, manual identification and outlining of cells was identified as being more accurate than applying Threshold calculations for cell measurement and enumeration (data are not shown). Physically processing of baked goods, including bread, results in the development of heterogeneous cellular structures that contribute to the mechanical behavior of the bread crumb (Zghal et al., 2002). AF and AOB had less cells per centimeter squared, and consisted of more cells between one and four millimeters squared, than OBA and OB, resulting in a less dense more open crumb (Table 2). OBA and OB were primarily composed of cells less than one millimeter squared and was significantly denser than the other breads. AF and AOB also had more cells between 4–7 and >7 mm^2^. Thus, almond flour contributes to a larger, softer crumb while oat bran fiber contributes to a finer, denser crumb.

**TABLE 2:** Range of cell size for ketogenic, gluten‐free bread. Values with common letters are not significantly different (*p* < .05)

Cell % Range	AF	AOB	OBA	OB
>1 mm^2^	37.7 ± 7.34 ^a^	56.8 ± 13.06^b^	75.1 ± 5.78^c^	80 ± 2.86^c^
1–4 mm^2^	45.2 ± 9.00^a^	33.0 ± 10.82^ab^	21.8 ± 5.52^bc^	17.7 ± 3.02^c^
4–7 mm^2^	10.7 ± 0.83^a^	7.5 ± 1.77^b^	1.9 ± 0.26^c^	0.9 ± 0.10^d^
<7 mm ^2^	6.4 ± 2.50^a^	2.7 ± 0.47^b^	1.2 ± 0.14^c^	1.4 ± 0.26^c^
Average density (cells/cm^2^)	13.2 ± 1.8^a^	16.8 ± 2.8^a^	25.3 ± 5.12^b^	27.8 ± 5.08^b^

### 3.2 Moisture and texture analysis

Results of texture and moisture analysis are presented in Table 3. AF was less moist than OB at 24 hr and less moist than all other breads after 72 hr. However, no significant difference remained at 120 hr as breads had equilibrated to the environment. Early difference may be attributed to the greater water binding and holding properties of oat bran fiber (Güler‐Akın et al., 2016; Krishnan et al., 1987; Liu et al., 2018; Sabanis et al., 2009). AF was less firm than OB at each time point and increases in firmness correlated with increases in oat bran fiber percentages. These results correlate with the findings during the evaluation of oat bran fiber in wheat rolls where particle size of oat bran fiber and hydrophilic associations were observed to impact binding of water (Kurek et al., 2016). It was interesting to note with the exception of OB, each variation decreased in firmness as breads aged. Breads that contain starches such as amylose and amylopectin typically go through the staling process and become more firm over time. In the absence of such starches, the opposite effect was observed. Additional studies are underway to explain these findings.

**TABLE 3:** Mechanical testing data for ketogenic, gluten‐free bread

Time	Attribute	Bread variation			
Hour		AF	AOB	OBA	OB
24	Moisture (%)	11.2 ± 0.54^Aa^	11.7 ± 0.42^ABa^	11.9 ± 0.19^ABa^	12.5 ± 0.05^Ba^
	Firmness (N)	15.7 ± 0.58^Aa^	18.1 ± 0.53^Ba^	19.8 ± 0.72^Ca^	24.9 ± 1.21^Db^
	Cohesion (g/s)	0.62 ± 0.012^Aa^	0.59 ± 0.013^Ba^	0.57 ± 0.011^Ca^	0.55 ± 0.013^Ca^
	Springiness (% recovery)	99.7 ± 0.21^Aa^	99.6 ± 0.23^Aa^	99.6 ± 0.28^Aa^	99.6 ± 0.30^Aa^
	Adhesion (g/s)	(−)21.5 ± 1.48^Aa^	(−)13.6 ± 2.32^Ba^	(−)13.9 ± 2.84^Ba^	(−)8.5 ± 2.27^Ba^
	Chewiness (N/g/s)	972.1^Aa^	1,074^ABa^	1,125.8^Ba^	1,375.6^Ca^
72	Moisture (%)	10.6 ± 0.31^Aa^	12.0 ± 0.33^Ba^	11.9 ± 0.40^Ba^	11.3 ± 1.33^Ba^
	Firmness (N)	12.9 ± 0.87^Ab^	15.3 ± 0.24^Bb^	17.2 ± 0.61^Cb^	27.6 ± 0.92^Da^
	Cohesion (g/s)	0.75 ± 0.009^Ab^	0.69 ± 0.014^Bb^	0.65 ± 0.019^Cb^	0.60 ± 0.006^Cb^
	Springiness (% recovery)	99.9 ± 0.21^Aa^	99.3 ± 0.19^Aa^	99.7 ± 0.34^Aa^	99.8 ± 0.20^Aa^
	Adhesion (g/s)	(−)29.6 ± 3.13^Ab^	(−)29.0 ± 5.34^Ab^	(−)28.4 ± 6.18^Ab^	(−)27.2 ± 3.84^Ab^
	Chewiness (N/g/s)	980.2^Aa^	1,050.3^ABa^	1,121.8^Ba^	1658.8^Cb^
120	Moisture (%)	11.3 ± 0.58^Aa^	11.8 ± 0.05^Aa^	11.9 ± 0.43^Aa^	11.3 ± 0.10^Aa^
	Firmness (N)	10.1 ± 0.28^Ac^	11.0 ± 0.35^Ac^	12.4 ± 0.24^Bc^	26.5 ± 1.25^Cab^
	Cohesion (g/s)	0.75 ± 0.014^Ab^	0.67 ± 0.013^Bb^	0.64 ± 0.015^Cb^	0.58 ± 0.004^Db^
	Springiness (% recovery)	99.9 ± 0.14^Aa^	99.9 ± 0.14^Aa^	99.5 ± 0.45^Aa^	99.9 ± 0.11^Aa^
	Adhesion (g/s)	(−)26.1 ± 7.96^Aab^	(−)56.1 ± 3.54^Bc^	(−)54.4 ± 1.70^Bc^	(−)28.8 ± 6.05^Ab^
	Chewiness (N/g/s)	761.2^Ab^	740.9 ^Ab^	791.7 ^Ab^	1,550.5^Bc^

Note

Upper case letters represent difference between bread variations and lower case letters represent differences of each variation at different times, Values with common letters are not significantly different (*p* < .05).

Higher percentages of almond flour correlated with increased cohesion scores, and AF was significantly more cohesive than AOB, which was higher than OBA, with OB being the least cohesive. Cohesion values increased in all varieties at 72 hr, and then no significant changes were observed at 120 hr. All breads exhibited extraordinary springiness and there were no significant difference between variations and time points. Typically, springiness is associated with positive interactions between gelatinized starches and gluten (Tegge, 1987). In the absence of both, it is proposed the high content of fat, proteins and fiber contribute to this resilience. Marco and Rosell (2008) identified protein enrichment of gluten‐free flours can contribute to increases in springiness (Marco & Rosell, 2008). The addition of fat compared to fat replacers also resulted in increased springiness in gluten‐containing breads (Scheuer et al., 2014). It is proposed the higher fat and protein content of these ketogenic, gluten‐free breads contributed to the resilience, and further studies to validate potential synergistic effects of this hypothesis are proposed.

At 24 hr, adhesion was significantly higher in AF, and at 72 hr, all breads showed slight increases in adhesion values, though no significant differences were observed. It was also apparent all breads appeared stickier visually and through tactile evaluation. However, after 120 hr, AF and OB were not different from each other while hybrids AOB and OBA were significantly more adhesive than homogeneous loaves. It should be noted, early texture profile analysis (TPA) described adhesiveness as a primary TPA parameter. However, more recently it is deemed as a secondary parameter and the value and validity of measuring adhesiveness via TPA is not considered accurate (Wee et al., 2018).

Chewiness of breads was correlated with increased percentages of oat bran fiber, with OB being the chewiest and AF being the least chewy at all evaluation points. All variations with almond flour decreased in chewiness over time, with significant differences being observed after 120 hr. As firmness is a driving influence of chewiness, this trend is not unexpected. Typically, cohesive values mirror chewiness as well. However, this study saw an inverse relationship between the two values which warrants further research.

### 3.3 QDA sensory evaluation

**FIGURE 3:**
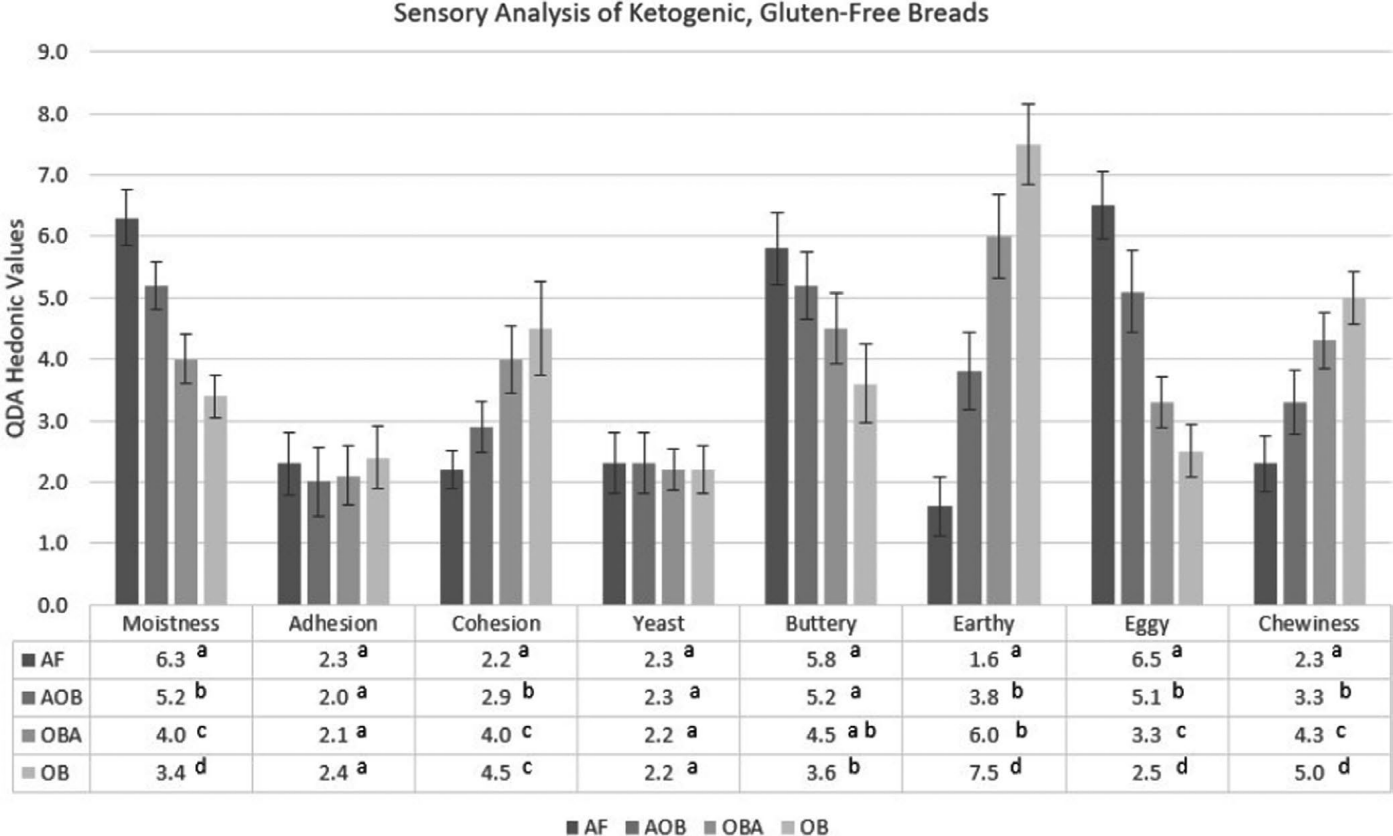
Sensory analysis of ketogenic, gluten‐free breads. Values with common letters are not significantly different (*p* < .05)

Bread characteristics from QDA evaluation are presented in Figure 3. Panel evaluation show moistness values between breads were significantly different. Variations containing higher levels of oat bran fiber were identified as less moist by the QDA panelists. As each bread formulation contained the same amount of nutritional yeast, it was not surprising the yeast flavor was not significantly different. Eggy flavor was more pronounced in breads containing higher percentages of almond flour. AF bread had the highest eggy scores and OBF was the least, with all formulas showing significant differences. Oat bran fiber can be considered the driving factor for earthy flavor, as AF was the least earthy, and as oat bran fiber percentages increased, earthy scores increased in statistically significant increments, with OBF being most earthy. Cohesive values were statistically significant between formulas, with AF identified as the least cohesiveness and OBF exhibiting the highest level of cohesiveness. Chewiness of breads mirrored the cohesiveness, with AF being least chewy and OBF being most chewy.

Bourne (1978) described the use of instrumental texture profile analysis extensively, using force, deformation, and work measurement to determine the texture parameters for hardness, fracturability, cohesiveness, adhesiveness, springiness, gumminess, and chewiness. Relationships between these attributes measured via mechanical means and sensory analysis are sometimes determined. There is strong support for correlations such as firmness or hardness between mechanical and sensory evaluations, however, as identified by Foegeding and Drake (2007), correlation between sensory attributes assessed by chewing, such as cohesiveness and adhesiveness do not align well with their mechanically measured counterparts (Foegeding & Drake, 2007). In their review of multiple food samples from candy to cheese and bakery products, Di Monaco et al. (2008) also ascertained an attribute such as hardness correlated well between sensory and mechanical testing methods, while mechanical cohesiveness endpoints were not predictive of their sensory counterparts (Di Monaco et al., 2008). Wee et al., (2018) also discussed parameters such as adhesion, cohesion, and chewiness, which are influenced by oral processing behaviors and do not always correlate with instrumental texture analysis (Wee et al., 2018).

Specific correlations between instrumental and sensory texture profiling can also vary among investigations as demonstrated when looking at the properties of cheese (Bryant et al., 1995; Drake et al., 1996, 1999). Multiple factors can explain the variations and include limitations in the mechanics of the tests; including discrepancies in changes in temperature as a result of chewing, variations between sample size and shapes, variations between rates of compression measure mechanically, and differences between mastication from person to person (Foegeding & Drake, 2007). Other studies have identified correlation between instrumental and sensory evaluation although correlation coefficients were very low (Matos & Rosell, 2012); and also identified how variance in oral processing influences food acceptability (Aguayo‐Mendoza et al., 2019; Liu et al., 2017). Associations between mechanical properties of foods and oral processing behaviors remain limited (Wee et al., 2018). Still, the involvement of variations in oral processing behaviors, including mastication force, and saliva secretion in sensory evaluations and the lack thereof in measuring parameters by mechanical means requires additional research before correlations between mechanical and sensory evaluations can be confidentially established.

### 3.4 Consumer acceptance

Consumers, informed and uninformed, showed a preference for AF and AOB breads over OBA and OB breads as demonstrated by overall acceptability and preference ranking scores. Additionally, consumers were least likely to purchase OB bread. AF was identified as having better flavor and moister than OB. Similar to the QDA panel, consumers rated AF higher in eggy flavor while OB was rated higher with earthy notes. Individual comments identified the earthy notes were less appealing for both informed and uninformed panelists. Panelists did not identify a significant difference in the appearance, aroma, texture, or chewiness of breads. (data are not shown).

## 4 CONCLUSION

Almond flour and oat bran fiber are suitable ingredients for ketogenic, gluten‐free breads. Higher ratios of almond flour to oat bran fiber resulted in a bread with better structure, flavor, and texture that was preferred by trained panelists and consumers. Higher levels of oat bran fiber resulted in a bread that was denser, more firm, and preferred less. Additional work is required to correlate sensory endpoints such as adhesiveness and cohesiveness, which involve mastication processing, with Texture Profile Analysis methods.

## CONFLICT OF INTEREST

The authors of this article certify that they have NO affiliations or involvement with any organization or entity related to the subject matter or materials discussed in this manuscript.

## AUTHOR CONTRIBUTION

**Rachel Gillespie:** Conceptualization (equal); Data curation (equal); Formal analysis (equal); Investigation (equal); Methodology (equal); Writing‐original draft (equal); Writing‐review & editing (equal). **Gene Ahlborn:** Conceptualization (equal); Data curation (equal); Formal analysis (equal); Funding acquisition (lead); Investigation (equal); Methodology (equal); Supervision (lead); Validation (equal); Writing‐original draft (equal); Writing‐review & editing (equal).

## Data Availability

The data that support the findings of this study are available from the corresponding author upon reasonable request.
